# Classification of parotid gland tumors by using multimodal MRI and deep learning

**DOI:** 10.1002/nbm.4408

**Published:** 2020-09-04

**Authors:** Yi‐Ju Chang, Teng‐Yi Huang, Yi‐Jui Liu, Hsiao‐Wen Chung, Chun‐Jung Juan

**Affiliations:** ^1^ Department of Electrical Engineering National Taiwan University of Science and Technology Taipei Taiwan; ^2^ Department of Automatic Control Engineering Feng Chia University Taichung Taiwan; ^3^ Department of Electrical Engineering National Taiwan University Taipei Taiwan; ^4^ Department of Medical Imaging China Medical University Hsinchu Hospital Hsinchu Taiwan; ^5^ Department of Radiology, School of Medicine, College of Medicine China Medical University Taichung Taiwan; ^6^ Department of Medical Imaging China Medical University Hospital Taichung Taiwan

**Keywords:** deep learning, head and neck, MRI, parotid gland tumor, transfer learning

## Abstract

Various MRI sequences have shown their potential to discriminate parotid gland tumors, including but not limited to *T*_2_‐weighted, postcontrast *T*_1_‐weighted, and diffusion‐weighted images. In this study, we present a fully automatic system for the diagnosis of parotid gland tumors by using deep learning methods trained on multimodal MRI images. We used a two‐dimensional convolution neural network, U‐Net, to segment and classify parotid gland tumors. The U‐Net model was trained with transfer learning, and a specific design of the batch distribution optimized the model accuracy. We also selected five combinations of MRI contrasts as the input data of the neural network and compared the classification accuracy of parotid gland tumors. The results indicated that the deep learning model with diffusion‐related parameters performed better than those with structural MR images. The performance results (*n* = 85) of the diffusion‐based model were as follows: accuracy of 0.81, 0.76, and 0.71, sensitivity of 0.83, 0.63, and 0.33, and specificity of 0.80, 0.84, and 0.87 for Warthin tumors, pleomorphic adenomas, and malignant tumors, respectively. Combining diffusion‐weighted and contrast‐enhanced *T*_1_‐weighted images did not improve the prediction accuracy. In summary, the proposed deep learning model could classify Warthin tumor and pleomorphic adenoma tumor but not malignant tumor.

## Graphical abstract

We used a two‐dimensional convolution neural network to conduct the segmentation and classifications of parotid gland tumors. The results indicated that the deep learning model with diffusion‐related parameters performed better than those with structural MR images. In summary, the deep learning model presented the potential to classify Warthin tumor and pleomorphic adenoma tumor, but not malignant tumor.

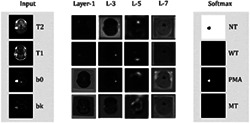

**Abbreviations**
2D: two‐dimensional
3D: three‐dimensional
ADC: apparent diffusion coefficient
ANTs: Advanced Normalization Tools
BraTS: Multimodal Brain Tumor Segmentation Challenge
CNN: convolution neural network
DWI: diffusion‐weighted imaging
EPI: echo‐planar imaging
MT: malignant tumor
NT: nontumor
PGT: parotid gland tumor
PMA: pleomorphic adenoma
WT: Warthin tumor
NPV: Negative Predictive Value
PPV: Positive Predictive Value

## 1 INTRODUCTION

Parotid gland tumor (PGT) is the most common type of salivary gland tumor. Major PGT types include pleomorphic adenoma (PMA), Warthin tumor (WT), and malignant tumor (MT). Determination of the type of PGT is crucial for clinical diagnosis and subsequent treatment. Imaging modalities, such as MRI and computed tomography, are useful to identify the location and size of PGTs. Fine‐needle aspiration biopsy is the primary method for identifying the tumor type, but its sensitivity is low (70%‐80%) for recognizing malignant PGTs.^1^, ^2^

MRI can be useful for tumor classification. For example, *T*_1_‐ and *T*_2_‐weighted images clearly present the texture of tumors, including areas of normal and lesion tissues.^3^ High‐grade malignant salivary gland tumors are distinguished on routine MR images by ill defined borders, cystic components, low *T*_2_ signal intensity, necrosis, and invasion of surrounding tissues. However, MRI is often unable to distinguish between benign and malignant salivary tumors.^4^, ^5^, ^6^ The apparent diffusion coefficient (ADC) derived from diffusion‐weighted imaging (DWI) has been shown to be associated with tumor cellularity, and MTs exhibit hyperintensity in DWI.^7^, ^8^ The ADC value of a PGT region is useful for differentiating between WTs and PMAs.^9^, ^10^, ^11^, ^12^ However, the mean ADC values of WTs and MTs are not significantly different.^8^, ^10^, ^13^ The ADC has a sensitivity of 50%‐60% in distinguishing MTs.^9^, ^14^ Therefore, identifying MTs through MRI remains a challenge.

Recently, deep learning methods, particularly convolution neural network (CNN)‐based models, have demonstrated effectiveness in image recognition tasks. CNN methods for pixel‐wise classification, also referred to as semantic segmentation, are now widely employed in computer‐vision applications, such as robotics and self‐driving cars.^15^, ^16^ The semantic segmentation method has also been used in MRI applications. For example, in the global competition of the Multimodal Brain Tumor Segmentation Challenge (BraTS),^17^, ^18^ researchers achieved an accuracy of more than 80% for the pixel‐wise classification of brain gliomas. In addition, deep‐learning‐based tumor segmentation and classification have been investigated for several cancers, including breast cancer,^19^, ^20^ liver tumor,^21^, ^22^, ^23^ and nasopharyngeal carcinoma.^24^ We hypothesize that the deep learning method on MRI data can also help detect and distinguish PGTs. In this study, we implemented a semantic segmentation method of multimodal MRI images for the segmentation of PGTs and classification of tumor types.

## 2 METHODS AND MATERIALS

### 2.1 The patient cohort and MRI protocol

The Institutional Review Board of Tri‐Service General Hospital approved the study and waived the requirement of written informed consent for this retrospective study. Eighty‐five consecutive patients with PGT (54 men and 31 women; age 49.6 ± 15.6 years) who underwent MRI examination were enrolled. Their PGTs were of the types WT (*n* = 27), PMA (*n* = 33), and MT (*n* = 25) according to histologic findings. All MRI examinations were performed on a 1.5 T MRI system (Signa HDx, GE Healthcare) with an eight‐channel neurovascular head‐and‐neck array coil. Before contrast administration, the scanning protocol included *T*_2_‐weighted and DWI sequences. Acquisition parameters for *T*_2_‐weighted imaging were slice orientation axial, *T*_R_ 3150 ms, *T*_E_ 77.3 ms, number of excitations 2, and slice number 32. Moreover, the single‐shot echo‐planar DWI with parameters (slice orientation axial, *T*_R_ 7000 ms, *T*_E_ 72.2 ms, number of excitations 4, slice number 18, and fat saturated) were acquired with diffusion gradients *b* = 0 and 1000 s/mm^2^ applied in each of three orthogonal directions. After contrast administration (gadolinium‐DTPA, 0.1 mmol/kg), we acquired *T*_1_‐weighted images by using fat‐saturated fast spin‐echo with the following parameters: slice orientation axial, *T*_R_ 616.7 ms, *T*_E_ 12 ms, number of excitations 0.5, and slice number 32. Thus, we collected datasets containing four MRI contrasts for each patient, namely *T*_2_ weighted, *T*_1_ weighted with contrast enhancement, *b*_0_ (DWI, *b* = 0 s/mm^2^), and *b*_1000_ (DWI, *b* = 1000 s/mm^2^).

### 2.2 Data conversion and image registration

After data acquisition, we collected all DICOM image files and sorted them by identifying DICOM tags. Next, the procedure converted files into NIfTI format files with the dcm2niix software (https://github.com/rordenlab/dcm2niix). We transferred them to a workstation for further processing. A board‐certified radiologist (CJJ), with more than 15 years of experience in head‐and‐neck MRI, manually outlined the region of the tumor on contrast‐enhanced *T*_1_‐weighted images and constructed another three‐dimensional (3D) volume with pixels labeled as 0, nontumor (NT; including background), 1, WT, 2, PMA, and 3, MT, according to histological records. Finally, the procedure saved the 3D volume with the tumor labels into another NIfTI file.

**FIGURE 1:**
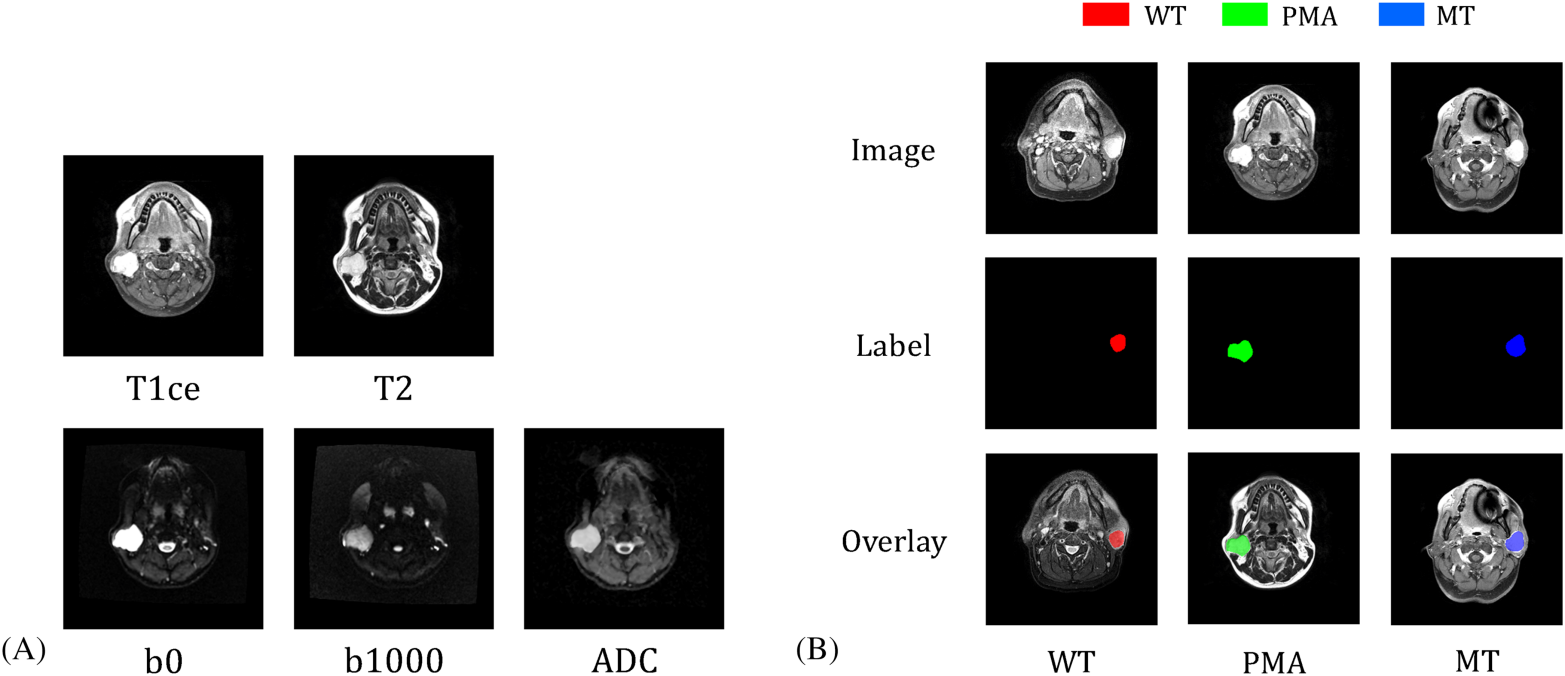
A, An example stack consisting of five types of MRI image. From left to right and top to bottom, they are contrast‐enhanced *T*_1_, *T*_2_, *b*_0_, *b*_1000_, and ADC images. The ADC map was reconstructed from *b*_0_ and *b*_1000_ images. We had 2726 stacks from 85 patients. B, Manually drawn regions of interest of the three types of tumor: NT areas are indicated in black, WT in red, PMA in green, and MT in blue. In the stored file, we used numbers 0 to 3 for NT, WT, PMA, and MT pixels, respectively

The subsequent step was to co‐register the four volumes by using the Advanced Normalization Tools (ANTs) software package (http://stnava.github.io/ANTs/). We registered the *T*_2_ volume to the contrast‐enhanced *T*_1_‐weighted volume. Subsequently, we used deformable registration to obtain the coordinate transformation between the *b*_0_ and *T*_2_ volumes and used the transformation to obtain the registered *b*_0_ and *b*_1000_ volumes. Using the obtained diffusion‐weighted volumes, we calculated ADC maps using the equation ADC = ln[(SI_0_/SI_1000_)]/1000, where SI_0_ and SI_1000_ are the signal intensities of the *b*_0_ and *b*_1000_ volumes, respectively.^25^ The ADC maps subsequently underwent median filtering with a 3 × 3 kernel. Therefore, we had six 3D volumes, including the contrast‐enhanced *T*_1_, *T*_2_, *b*_0_, *b*_1000_, ADC, and tumor label volumes. They were presented as $\hat{T_{1}}$, $\hat{T_{2}}$, $\hat{b_{0}}$, $\hat{b_{1000}}$, $\hat{\mathrm{ADC}}$ and $\hat{\mathrm{SEG}}$, respectively. The matrix size of each volume was 256 × 256 × 32. For the upcoming deep learning procedures, we normalized the pixel values of $\hat{T_{1}}$, $\hat{T_{2}}$, $\hat{b_{0}}$, and $\hat{b_{1000}}$ based on the maximum intensity of each volume. Finally, we extracted two‐dimensional (2D) slices from the 3D volumes for further usage in the deep learning training procedures. For every slice, we merged $\hat{T_{1}}$, $\hat{T_{2}}$, $\hat{b_{0}}$, $\hat{b_{1000}}$, and $\hat{\mathrm{ADC}}$ data into a 256 × 256 × 5 matrix. This matrix was termed a five‐channel “stack.” We collected 2726 stacks from the 85 patients, including 463 stacks covering PGTs and 2263 stacks without PGTs. Figure 1A shows an example stack of the five modalities of MRI. Figure 1B presents the examples of the manually outlined region of the three tumor types (red, WT, green, PMA, and blue, MT). Because of the restriction of the input layer of the implemented neural network, which will be discussed later, the input stack size was fixed to a four‐channel stack (256 × 256 × 4). To compare the relation between classification accuracy and MR contrasts, we generated the following types of four‐channel stack: sT2 combining four identical images ( $\hat{T_{2}}$, $\hat{T_{2}}$, $\hat{T_{2}}$, and $\hat{T_{2}}$), sT1 combining ( $\hat{T_{1}}$, $\hat{T_{1}}$, $\hat{T_{1}}$, and $\hat{T_{1}}$), sT1T2 combining ( $\hat{T_{1}}$, $\hat{T_{1}}$, $\hat{T_{2}}$, and $\hat{T_{2}}$), sDWI consisting of (zeros, $\hat{b_{0}}$, $\hat{b_{0100}}$, and $\hat{\mathrm{ADC}}$), sALL consisting of ( $\hat{T_{1}}$, $\hat{T_{2}}$, $\hat{b_{0}}$, and $\hat{b_{1000}}$), and sALL2 consisting of ( $\hat{T_{1}}$, $\hat{T_{2}}$, $\hat{b_{1000}},\text{and}\;\hat{\;\mathrm{ADC}}$).

### 2.3 Deep learning: U‐Net and transfer learning

We used 2D U‐Net for pixel‐wise tumor classification.^26^ The network consisted of encoding and decoding paths with convolutional blocks. Each of the blocks consisted of a 3 × 3 convolution layer followed by a rectified linear unit and a dropout layer. In the encoding path, the output of each block was down‐sampled with a max‐pooling operation with a stride of 2. In the decoding path, the input of each block was concatenated with the corresponding feature maps obtained in the encoding path, and the output of each block was up‐sampled using a transpose convolution. The final output layer of the U‐Net was connected to a multiclass softmax classifier.

To initialize U‐Net, we used transfer learning, which refers to transferring network weights from a pretrained model to another model. In general, the pretrained models are trained with immense numbers of datasets. With the same architecture of the deep learning network, the weights of the pretrained model can be utilized as the initial values of the weights for the new model. Because the weights are linked to the process of extracting and filtering features, most deep learning machines are specialized in a particular field or task. Therefore, we adapted a method that won the third prize in BraTS 2017 to produce the pretrained model.^27^ In that model, a four‐channel input layer was implemented, and the U‐Net was pretrained with four types of brain MR image (ie *T*_2_, FLAIR, *T*_1_, and contrast‐enhanced *T*_1_) and three tumor labels (1, necrotic and nonenhancing tumor, 2, peritumoral edema, and 3, gadolinium‐enhanced tumor). The training set of BraTs 2017 included 285 patients with gliomas. The matrix size of the BraTs 2017 dataset was 240 × 240 × 155. We extracted 155 2D slices from each dataset and interpolated their matrix size into 256 × 256. The total number of images and steps for training the BraTS model were 155 × 285 = 44 175 and 600 000, respectively.

After constructing the pretrained model, we transferred its weights to initialize the training procedure in our current study for classifying PGTs. The training parameters of U‐Net were the following: optimizer, Adam, batch size, 6 or 8, loss, cross‐entropy, beta of L2 regularization, 10^−7^, and training steps, 10 000. We employed image augmentation methods, including random image up‐down and left‐right flipping, rotation (ranging from 15° to −15°), and contrast adjustments (ranging from 0.6 to 1.4), in the training procedures to enhance the variability of the images. The U‐Net was implemented with the TensorFlow framework (v1.8) under the Python (v3.6) environment and was trained on a home‐built workstation with a 1080 GPU (1080 Ti, Nvidia Corporation, Santa Clara, CA, USA).

### 2.4 Cross‐validation, prediction, and performance assessment

We distributed the 2726 stacks into three groups by using stratified random sampling to conduct a threefold cross‐validation of the U‐Net model.^26^ All the stacks of one patient were dispatched into the same group, and every group had a proportional allocation of the three tumor types. The number of patients in the three groups was (WT 9, PMA 8, WT 8), (9, 8, 8), and (9, 8, 9), respectively. We performed eight trials of random sampling and U‐Net training. After the training stage, we built U‐Net models for the pixel‐wise classification of 2D MR images. Because the PGT datasets were 3D volumes with a matrix size of 256 × 256 × 32, we split each 3D volume into 32 images, obtained the model inferences of the images, and then merged them back into a 3D volume. This volume is termed the predicted $\hat{\mathrm{SEG}}$. The matrix size of the predicted $\hat{\mathrm{SEG}}$ was the same as the input volume (ie 256 × 256 × 32), and pixel values presented classification results (ie 0 for background, 1 for WT, 2 for PMA, and 3 for MT). Subsequently, for each predicted $\hat{\mathrm{SEG}}$, we counted the pixel numbers of three tumor types in it, identified the tumor type with the largest pixel number, and unified the tumor category for all slices. Finally, we stored the predicted $\hat{\mathrm{SEG}}$ in a NIfTI file format. We then evaluated segmentation results by using Dice coefficients and calculated the accuracy of tumor classification results.

### 2.5 Comparing training schemes: transfer learning and input batches

During the preliminary investigation stage, we evaluated four training schemes to determine a scheme that optimized the tumor classification performance. In Scheme 1, the input batch size was six, and transfer learning was not applied. The training procedure randomly selected six stacks from the training stacks containing all three PGTs. Scheme 2 was the same as Scheme 1 but with transfer learning. Scheme 3 was the same as Scheme 2 except that the input batch was not a random mix of three tumor types but comprised exactly two WT, two PMA, and two MT stacks. In Scheme 4, transfer learning was applied, and all stacks, including both tumor and NT stacks, were used to train the U‐Net model. The batch size was eight stacks—two WT, two PMA, two MT, and two NT stacks. To select the optimized training scheme, we trained the U‐Net model by using the sDWI stacks and the four schemes, and the training scheme that yielded the best results was then chosen to evaluate the segmentation and classification performance.

## 3 RESULTS

### 3.1 Visualization of training procedures

**FIGURE 2:**
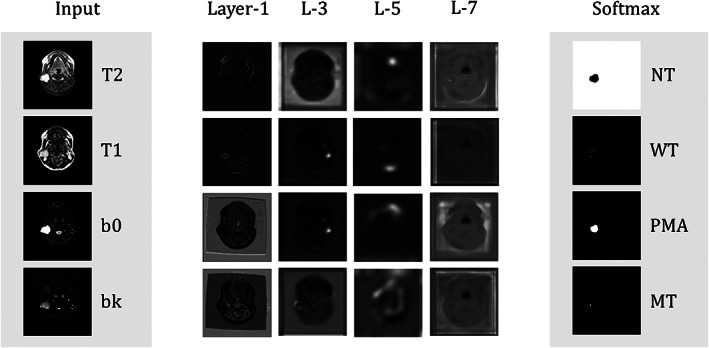
Visualization of the output images of the intermediate and softmax layers of the sALL U‐Net model. Layers 1 to 5 are in the encoding path, and output images are down‐sampled with max‐pooling to reduce feature numbers. The pixel values of the output images of the softmax layer present the probability distribution (white for 1, black for 0) of four categories (ie NT, WT, PMA, and MT). As an example, the large region of white color in the upper‐right image (the NT image from softmax output) means that these white pixels likely belong to the NT category, except for the small dark region, likely belonging to a pleomorphic tumor (cf. the PMA image from softmax output)

Figure 2 displays intermediate results obtained during the training process of sALL. The first column presents all input images, including $\hat{T_{1}}$, $\hat{T_{2}}$, $\hat{b_{0}}$, and $\hat{b_{k}}$. The following three columns demonstrated the outputs of different convolutional layers. The features were extracted layer by layer in the encoding path, and the output of the fifth layer was found to have the lowest spatial resolution. Next, an up‐sampling (decoding) path was started. The output of the seventh layer demonstrated a higher spatial resolution than that of the fifth layer. The network generated softmax values for each class of each pixel. The value presented the probability distribution of the four categories (ie NT, WT, PMA, and MT) for each pixel.

### 3.2 Training scheme comparison

**FIGURE 3:**
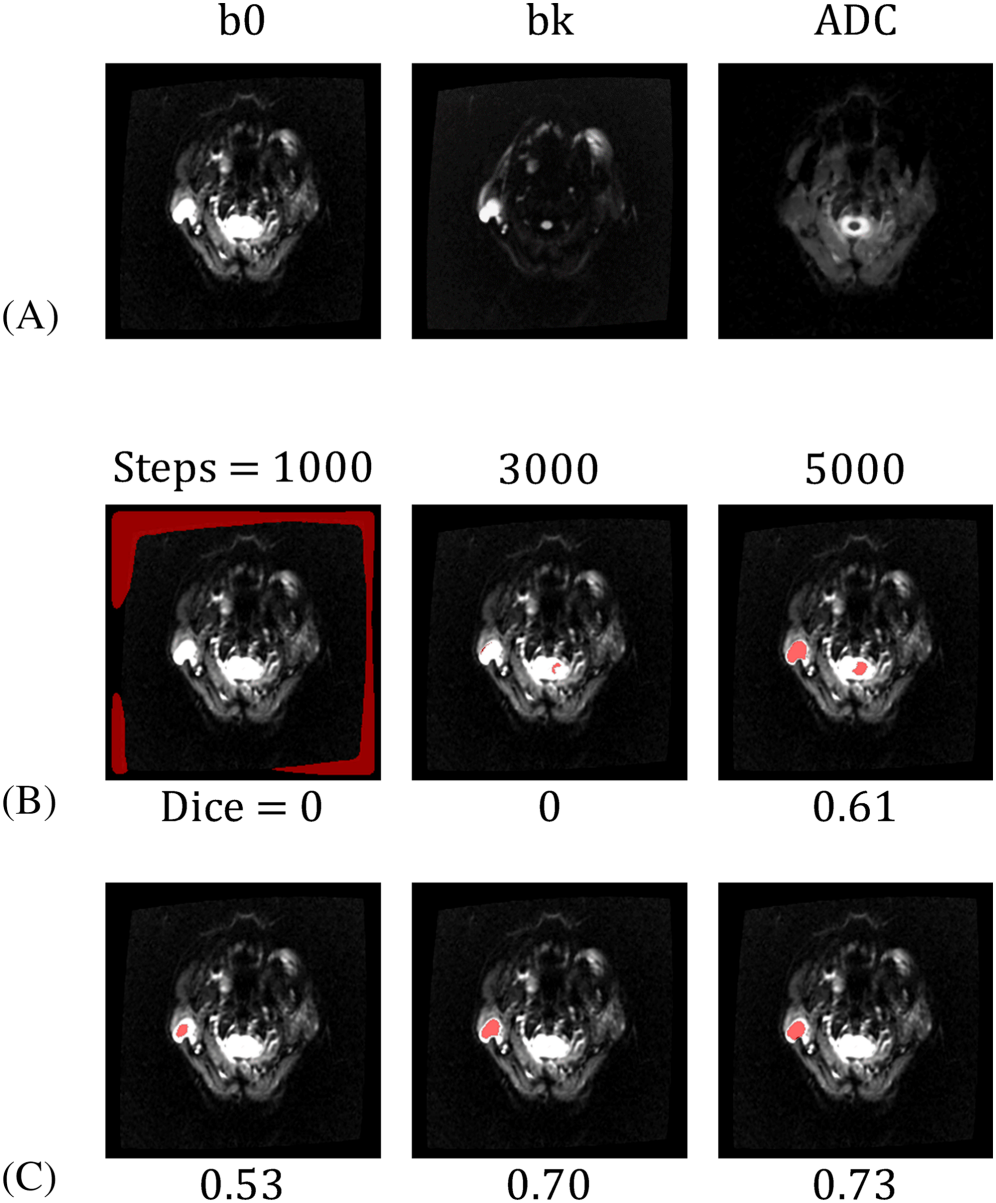
Demonstration of the PGT segmentation of an image containing a WT on the right side. The input is an sDWI stack (A), and WT regions are predicted using models trained with Scheme 1 (B) and Scheme 2 (C). The WT regions are presented as red pixels overlaying the ADC images. After 1000, 3000, and 5000 training steps, the WT region became progressively more accurate. The number beneath each image is the Dice coefficient between the predicted and actual tumor regions. This example demonstrates the advantage of using transfer learning in Scheme 2

Figure 3 compares Schemes 1 and 2. Figure 3A shows the input DWI stack. This patient had one WT in the right parotid gland. Figure 3B presents predicted $\hat{\mathrm{SEG}}$ after different training steps for Scheme 1 (upper row) and Scheme 2 (lower row), respectively. The tumor was clearly outlined after 5000 training steps for Scheme 1 and 1000 for Scheme 2. After 5000 steps, the Dice coefficient of the Scheme 2 result was considerably higher than that of Scheme 1. This suggested that applying transfer learning in Scheme 2 not only accelerated the convergence of U‐Net optimization but also improved the prediction accuracy. Table 1 presents the group analysis of the four training schemes for the recognition of PGTs. Scheme 1 exhibited the poorest prediction performance; Scheme 4 displayed the best prediction results, with accuracy values for WT, PMA, and MT being 0.81, 0.76, and 0.71, respectively.

**TABLE 1:** Results of the methods used for the identification of PGTs

	Batch size/input data	Transfer	Accuracy of each scheme		
			WT (*n* = 27)	PMA (33)	MT (25)
Scheme 1	6, random (WT, PMA, MT)	✗	0.73 ± 0.05	0.69 ± 0.04	0.64 ± 0.04
Scheme 2	6, random (WT, PMA, MT)	✓	0.77 ± 0.03	0.74 ± 0.01	0.69 ± 0.04
Scheme 3	6, WT × 2, PMA × 2, MT × 2	✓	0.78 ± 0.02	0.74 ± 0.03	0.67 ± 0.03
Scheme 4	8, WT × 2, PMA × 2, MT × 2, NT × 2	✓	0.81 ± 0.02	0.76 ± 0.02	0.71 ± 0.04

### 3.3 Comparing multimodal MRI

**FIGURE 4:**
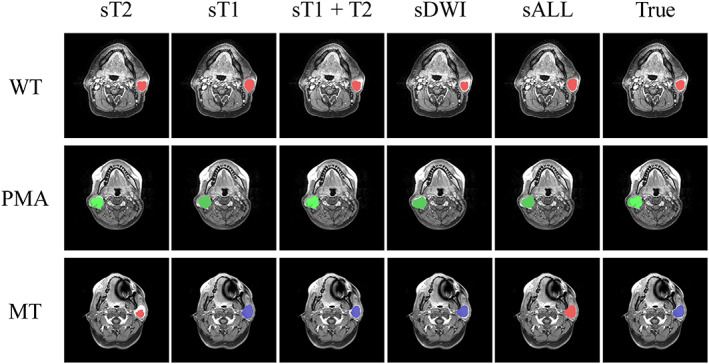
Three selected slices of the predicted PGT regions (red, WTs, green, PMAs, and blue, MTs). For WT and PMA slices, the U‐Net models trained using different stacks correctly classified the tumor class. However, for the MT example, the sT2 and sALL models misidentified MTs as WTs

Next, we used the Scheme 4 method to obtain models with six types of stack. Each model underwent a threefold cross‐validation with eight trials, with 680 (85 patients × 8 trials) predicting $\hat{\mathrm{SEG}}$ results for each type of stack. Figure 4 demonstrates predicted results obtained from three patients having WT, PMA, or MT. For the patients with WT or PMA, the results of the five stack types were comparable to the true label. For the patient with MT, the segmentation results of all the types of stack, except sT2, were close to the correct label. However, only results obtained from sT1, sT1T2, and sDWI classify PGT into the correct category (ie MT, blue color). Table 2 presents the group statistics of the recognition of PGTs. For segmentation results, the PGT region obtained using sALL produced the highest average Dice coefficient (0.48 ± 0.01). For tumor classification, PGT types predicted using sDWI yielded the highest average accuracy. The group analysis revealed that the performance of sT2 was the poorest. Combining DWI and structural images did not improve the outcome. Among all models, classifying WTs exhibited the highest accuracy. Table 3 presents the complete results of classification using sDWI. The sensitivity of WT, PMA, and MT was 0.83, 0.67, and 0.33, respectively, suggesting that the obtained U‐Net model was not sensitive to classify MTs.

**TABLE 2:** Results of the identification of PGTs using various combinations of MR images

Input	Dice	Accuracy of each type		
		WT (*n* = 27)	PMA (33)	MT (25)
sDWI	0.46 ± 0.03	0.81 ± 0.02	0.76 ± 0.02	0.71 ± 0.04
sT1	0.44 ± 0.01	0.79 ± 0.02	0.66 ± 0.03	0.68 ± 0.04
sALL	0.48 ± 0.01	0.75 ± 0.04	0.70 ± 0.03	0.62 ± 0.03
sALL2	0.40 ± 0.02	0.83 ± 0.03	0.67 ± 0.01	0.68 ± 0.02
sT1T2	0.42 ± 0.02	0.72 ± 0.04	0.65 ± 0.04	0.62 ± 0.04
sT2	0.38 ± 0.02	0.67 ± 0.05	0.63 ± 0.04	0.62 ± 0.04

**TABLE 3:** Classification results of sDWI

	Accuracy	Sensitivity	Specificity	PPV	NPV
WT	0.81 ± 0.02	0.83 ± 0.03	0.80 ± 0.03	0.66 ± 0.03	0.91 ± 0.01
PMA	0.76 ± 0.02	0.62 ± 0.07	0.84 ± 0.03	0.72 ± 0.02	0.78 ± 0.03
MT	0.71 ± 0.04	0.33 ± 0.07	0.87 ± 0.04	0.53 ± 0.12	0.76 ± 0.02

## 4 DISCUSSION

In this study, we describe a fully automatic system for the detection and classification of PGTs. We used a 2D U‐Net CNN for multiclass segmentation. To investigate a suitable training procedure, we proposed and compared four training schemes for U‐Net. We found that transfer learning and manipulations of training batches gradually improved the classification accuracy. Unlike Scheme 1, we applied transfer learning in Scheme 2, which improved performance; this suggests that the network weights pretrained with 44 175 stacks in the BraTs dataset may lead the U‐Net model closer to the optimal solution because filters for the detection of the brain and PGTs in convolutional layers could be similar. In Schemes 3 and 4, the training batch for the forward path of U‐Net was a fixed structure. Each batch in Scheme 3 comprised two stacks of each tumor type, with class balance maintained during the training stage. Although this setup improved the classification accuracy, a nonignorable number of NT pixels were misclassified into PGTs (ie false positives). In Scheme 4, we added two stacks generated from image slices without PGTs in each batch. The performance of Scheme 4 was the best. Thus, we fixed the training procedure to that used for Scheme 4 and kept exploring the model efficiency with various combinations of MRI images to identify PGT types.

We used six types of stack as input to the U‐Net model and obtained the corresponding models. The Dice coefficient results revealed that sALL and sDWI models produced better segmentation results than the other models. The sDWI model outperformed the others in accuracy. Among the stacks consisting of only structural images, the sT1 model performed better than the sT2 or sT1T2 models. As for sALL and sALL2, we assembled it with all available MR modalities, such as *T*_1_, *T*_2_, and DWI, and assumed that the U‐Net training procedure could derive the optimal combination of all MR information. However, neither model was better than the sDWI model. This could be for two reasons: image registration and data size. For example, the four‐channel sALL stack (matrix size 256 × 256 × 4) was constructed from four images ( $\hat{T_{1}}$, $\hat{T_{2}}$, $\hat{b_{0}}$, and $\hat{b_{1000}}$), with the assumption of multichannel deep learning that all the channels were aligned pixel by pixel. Although we used deformable registration to amend the misalignment between spin‐echo‐based structural images and echo‐planar imaging (EPI)‐based DWI images, residual image distortion along the phase‐encoding direction in DWI images was inevitable, thereby impairing registration precision. The misregistration between channels in the sALL‐based U‐Net model could have reduced the accuracy of PGT recognition. We merged all MR information in the input layer and tested whether the training procedure could select dominating image channels and exclude less useful ones. In theory, if the training procedure achieves the optimal solution, the sT1T2 model should be at least comparable to the sT1 model under equal computation power. However, our limited data size (2726 stacks) could have restricted model optimization, and more information did not produce better results.

Among all the models investigated in this study, the sDWI model provided the best PGT classification results. The accuracy results were 0.81, 0.76, and 0.71 for WT, PMA, and MT, respectively. The classification performance of WT is comparable to that reported in a previous study, which required sex and age information in addition to MRI images.^12^ The sensitivity results were 0.83, 0.63, and 0.33 for WT, PMA, and MT, respectively. The results suggest that our current model was insensitive to MTs. This critical limitation originates from the fact that malignant PGTs in humans are related to deeper structures, such as the parapharyngeal space, adjacent muscles, and bony tissues, which are not clearly presented in MRI.^28^ In addition, among WT, PMA, and MT, the ADC values of PMAs are higher than those of MTs and WTs. In previous studies on PGTs, ADC values were used to differentiate PMA or WT.^10^, ^12^, ^29^ However, MTs with increased cellularity and WTs containing lymphoid tissues both present lower ADC values. Consequently, when only ADC values are used, an overlap of ADC values between WTs and MTs impedes the effective differentiation of MTs. Studies have suggested that fraction anisotropy values obtained using diffusion tensor imaging^30^ and the wash‐out pattern of dynamic contrast imaging^31^ improve diagnostic accuracy. Additional studies should combine them into the deep learning framework to potentially improve sensitivity to distinguish MTs.

Compared with radiomics‐based machine learning methods,^32^, ^33^ the proposed algorithm both outlines the tumor region and identifies the tumor type. It does not need region of interest drawing once the training is completed. It is therefore fully automatic and beneficial in the clinical setting. Furthermore, the procedures for the generation of image features are different in the machine and deep learning methods. CNN learns image features according to the training dataset, whereas machine learning methods use predefined features. If the training procedure of a deep learning method achieves the optimal solution with sufficient training data, the obtained image features could outperform predefined ones. However, features generated by the “black box” deep learning methods are more difficult to interpret than predefined features (eg gray‐level texture features in radiomics) in machine learning models. If explainable features are desirable, machine learning algorithms may be used for this application instead of deep learning.

One study limitation is the small data size, which hampers the optimization of the large segmentation network. Although transfer learning improves the classification accuracy, deep learning models with a larger dataset are warranted. Another study limitation is the alignment of DWI, *T*_1_‐weighted, and *T*_2_‐weighted images. Although we retrospectively performed deformable registration of all images, the residual misregistration may have reduced the classification performance. Reducing EPI distortion or acquiring all images with the same type of MR sequence, such as the multishot EPI,^34^ may be a solution.

In summary, we assessed the PGT classification performance of a U‐Net method combined with multimodal MRI. The U‐Net model based on DWI information outperformed contrast‐enhanced *T*_1_‐ and *T*_2_‐weighted images. Combining all available modalities did not improve accuracy. The U‐Net model could simultaneously outline the tumor region and identify the tumor type. The U‐Net model can be practical to use in the clinical setting to detect WTs and PMAs, but it is not sensitive for MTs.

## CONFLICTS OF INTEREST

The authors declare that they have no financial interests or potential conflicts of interest related to the research described in this paper.

## Data Availability

The datasets generated or analyzed during the current study are available from the corresponding author on reasonable request.
